# A novel classification predicts prognosis and drug sensitivity in osteosarcoma based on alterations in gene sets

**DOI:** 10.18632/aging.205614

**Published:** 2024-02-28

**Authors:** Shuxi Ye, Xiaopeng Wang, Rongchun Chen

**Affiliations:** 1 Department of Spine Surgery, Ganzhou People’s Hospital, Ganzhou, Jiangxi, China

**Keywords:** osteosarcoma, pathways, tumor microenvironment, drug sensitivity

## Abstract

Osteosarcoma is a cancer originating in the bone cells, specifically in the osteoblasts. Previous studies mainly focused on particular molecules but the whole pathway network. We comprehensively analyzed the enrichment score of each signal pathway and identified a novel classification by 20 machine learning algorithms. Furthermore, differences in tumor immune infiltration cells and drug sensitivity were compared in low and high groups. We identified a model consisting of four signaling pathways that predict the prognosis and the immune status of the tumor microenvironment and drug sensitivity in osteosarcoma patients. The novel classification may be used in clinical applications to predict prognosis and drug sensitivity.

## INTRODUCTION

Osteosarcoma is a type of cancer that originates from the bone cells, specifically in the osteoblasts, which are responsible for bone formation [1]. It is the most common primary malignant tumor of the bone, typically affecting children, adolescents, and young adults. This form of cancer has a strong predilection for long bones, such as the arms and legs, but it can also occur in other bones [2, 3]. The exact cause of osteosarcoma is still unclear. However, certain risk factors have been identified, including genetic predisposition, previous radiation therapy, and certain hereditary conditions [4, 5]. Osteosarcoma can also occur secondary to other bone disorders, such as Paget’s disease [6].

One of the hallmark features of osteosarcoma is its aggressive nature and high potential for metastasis. It tends to spread to distant sites, most commonly the lungs, before symptoms become apparent. The initial symptoms may include localized pain, swelling, and limited range of motion in the affected area. The tumor can weaken the bone as it grows, leading to fractures or pathologic fractures, which occur with minimal trauma. Diagnosis of osteosarcoma involves a combination of imaging studies, such as X-rays, CT scans, and MRI scans, along with a biopsy to confirm the presence of malignant cells. Histological examination of the biopsy sample helps determine the tumor grade, which is essential for assessing the aggressiveness of the cancer. Treatment for osteosarcoma often involves a multidisciplinary approach. The mainstay of treatment is surgical resection of the tumor, which aims to remove the tumor along with a margin of healthy tissue to reduce the risk of local recurrence [2, 7]. The operation may be followed by chemotherapy, administered either before or after surgery. Chemotherapy is crucial in targeting any microscopic metastases that may be present. In some cases, radiation therapy may also be used as an adjunct to surgery and chemotherapy. The prognosis for osteosarcoma has improved significantly over the years, thanks to advances in treatment strategies. Factors influencing prognosis include the size and location of the tumor, the presence of metastasis, the response to chemotherapy, and the patient’s overall health. With appropriate treatment, the five-year survival rate for localized osteosarcoma is around 60–75%, but it decreases to approximately 15–30% if metastasis has occurred.

In recent years, there has been a growing interest in identifying and validating biomarkers for osteosarcoma. Biomarkers are biological molecules or characteristics that can be measured and used to indicate the presence or severity of a disease, predict its progression, or evaluate the response to treatment. They can be found in various biological samples, including blood, tissue, and urine, and derived from proteins, genes, or other molecules. Several biomarkers have been proposed for osteosarcoma, with potential applications in diagnosis, prognosis, and treatment response prediction. Some of the most promising biomarkers for osteosarcoma. Alkaline phosphatase (ALP): Elevated serum ALP levels have been associated with a higher tumor burden and poorer prognosis in osteosarcoma patients. Pre-treatment ALP levels may also help predict response to chemotherapy [8, 9]. Lactate dehydrogenase (LDH): Similar to ALP, increased serum LDH levels have been linked to a higher tumor burden and worse prognosis in osteosarcoma patients. Pre-treatment LDH levels may also help predict chemotherapy response [10, 11]. Circulating tumor cells (CTCs): The presence of CTCs in the blood of osteosarcoma patients has been associated with a higher risk of metastasis, and their detection may be useful for monitoring disease progression and response to treatment [12–14]. MicroRNAs (miRNAs): These small, non-coding RNA molecules have been implicated in the regulation of various cellular processes, including proliferation, differentiation, and apoptosis. Dysregulated miRNA expression has been observed in osteosarcoma, and specific miRNAs have been proposed as potential diagnostic and prognostic biomarkers, as well as therapeutic targets [15]. Genetic alterations: Mutations in genes such as TP53, RB1, and RECQL4 have been associated with an increased risk of osteosarcoma. Additionally, chromosomal abnormalities, such as amplifications or deletions, have been identified in osteosarcoma tumors and may serve as potential prognostic markers [16, 17].

While these biomarkers promise to improve the diagnosis, prognosis, and treatment of osteosarcoma, most of them do not predict patient prognosis and drug sensitivity. As the tumorigenesis of osteosarcoma is the consequence of altering multi-genes and multi-pathways, we used multiple machine learning algorithms to identify a pathways-related signature associated with the prognosis of osteosarcoma. Further analysis revealed that the signature can be used as the biomarker of drug sensitivity in patients with osteosarcoma.

## METHODS

### Data acquirement and processing

**Figure 1:**
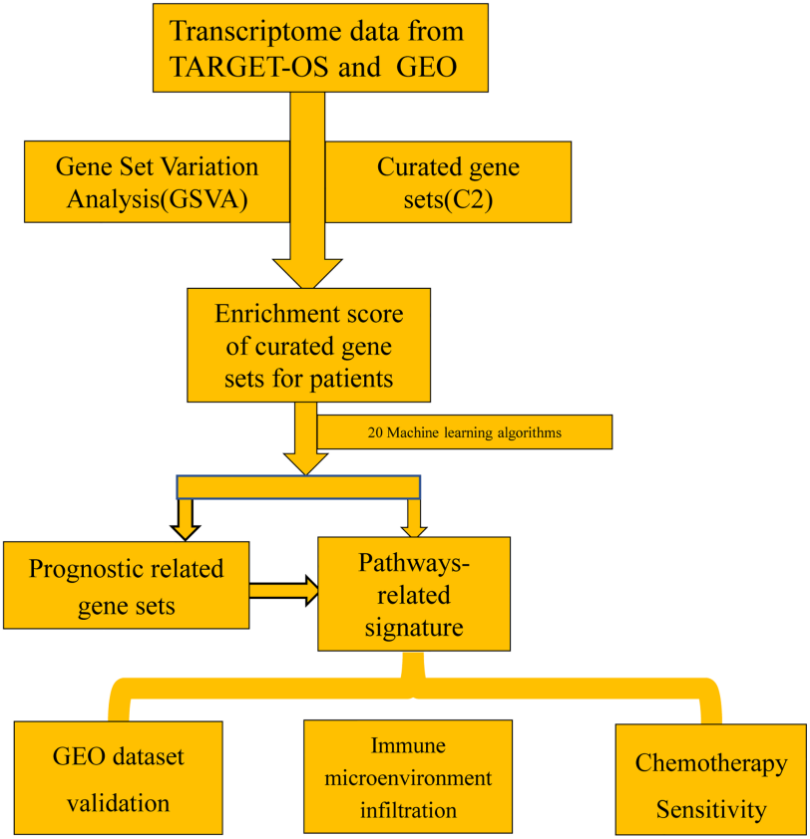
The main workflow of the study.

The Therapeutically Applicable Research to Generate Effective Treatments (TARGET) program aims to identify the molecular alterations that underlie juvenile malignancies by multi genomic approaches. The transcriptome RNA sequence data from TARGET was downloaded by XENA datasets [18]. Our study comprised 85 patients with comprehensive clinical information (e.g., age, gender, survival status, survival time). In order to increase the credibility of the study, we conducted a validation with an external dataset from the GEO dataset with GSE21257, which includes 53 patients [19]. The main workflow is presented in Figure 1.

### Gene set variation analysis and prognosis related pathways

Previous studies have focused on one aspect of sequencing data (e.g., mRNA, miRNA, mutation), whereas tumorigenesis is a multigene, multi-signaling pathway process. Therefore, we first calculated the enrichment scores of multiple signaling pathways for each patient by R package of “GSVA” [20]. Prognosis-related signaling pathways were identified based on clinical follow-up information by coxph function of R package “survival” [21].

### Machine learning and feature selection

An increasing number of studies have shown that machine learning algorithms can be used for feature selection and outperform traditional algorithms. In this study, we apply 20 algorithms or parameters for feature screening. Random survival forests (RSF) were applied to extend random forest to the setting of right-censored survival data. The R package of “randomForestSRC” was used to conduct the random forest algorithms [22]. The stochastic gradient boosting strategy was used by the R package of “gbm” [23]. The stepCOX algorithm consists of two main steps: the first step is a proportional COX regression, and the second step screens the best COX model by the step function. Enet (Elastic Net) is an overlay approach and a linear regression model that combines L1 and L2 regularisation. Elastic Net allows for simultaneous feature selection and parameter control, avoiding some of the limitations of traditional regularisation methods. Enet is implemented through the “glmnet” package [24]. CoxBoost fits Cox proportional risk models by means of a boost method based on fractional likelihood. It is particularly suitable for models with a large number of predictors and allows for forced covariates with uninitialized parameter [25]. Lasso regression (Least absolute shrinkage and selection operator) and Ridge regression were conducted using the R package of glmnet. plsRcox is used to build a predictive model that can predict the survival of a subject based on multiple characteristics of the subject. In this model, PLS (Partial Least Squares) method and regression method are combined to improve the accuracy of prediction [26]. The survivalSVM algorithm integrates survival analysis and support vector machines for regression analysis. ‘Superpc’ is useful for high-dimensional data [27].

The C-index was employed to determine the discriminatory power of various models. In this study, var.select function from R package of varSelRF was used to determine the variables used for subsequence analysis.

### Risk stratification analysis

To make the model more straightforward in clinical practice, we used a multivariate Cox proportional risk model to construct the signature with select features identified by the machine learning method. The risk score was calculated for each patient. Patients were divided into high- or low- groups based on the risk score.

### Differences in tumor microenvironment between high- and low-risk groups

The tumor microenvironment was enumerated by R packages of Xcell [28] and IOBR [29], which had been reported to outperform other methods in comparison to cytometry immunophenotyping based on the gene signature method.

### Drug sensitivity analysis between high- and low-risk groups

The relative drug sensitivity was calculated by R package of oncoPredict with the function of calcPhenotype. oncoPredict predicts sensitivity values for a wide range of tumor-related drugs based on Genomics of Drug Sensitivity in Cancer with 198 compounds or drugs.

### Statistical analysis

All the data were processed by R 4.3.0. Wilcox or unpaired Student’s *t*-test for the continuous variable and chi-square test for dichotomous variables were applied to determine the statistical significance of differences between high- and low-risk groups. Unless otherwise noted, 2-sided and *p* < 0.05 were used to determine statistical significance for all tests.

## RESULTS

### Identification of survival-associated pathways in patients

Our study included 85 patients with complete clinical information in TARGET datasets and 53 patients in GEO dataset were included.

The TARGET dataset was employed to identify the prognosis-related pathways. This analysis determined 486 pathways as the survival-related signal pathways in osteosarcoma. To facilitate subsequent calculations as well as to improve the accuracy of the predictions, we incorporated the top 20 signaling pathways into the machine learning model.

### Comparison of multiple machine learning algorithms

**Figure 2:**
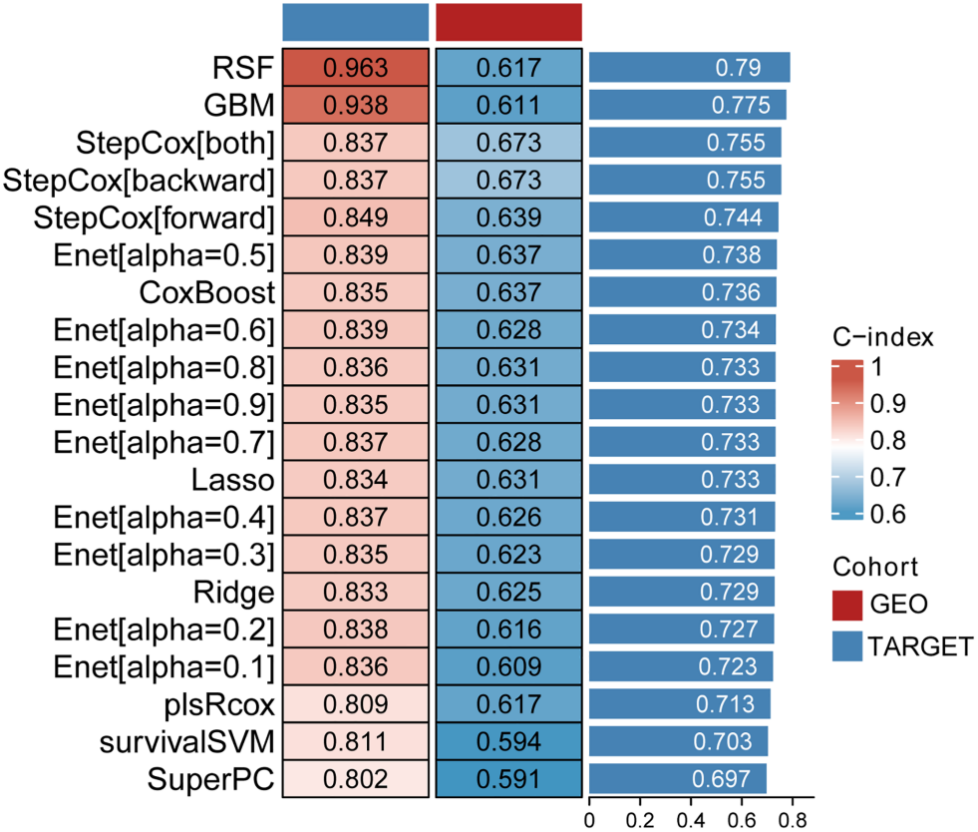
The C-index of 20 machine learning algorithms.

The top 20 signaling pathways with the smallest *p*-values were included in the machine learning model for evaluation. Variables were screened by 20 machine learning algorithms (“survivalSVM”, “Ridge”, “SuperPC”, “Enet (alpha = 0.1)”, “Enet (alpha = 0.2)”, “Enet (alpha = 0.3)”, “Enet (alpha = 0.4)”,0 “Enet (alpha = 0.5)”, “CoxBoost”, “Enet (alpha = 0.6)”, “Enet (alpha = 0.8)”, “Enet (alpha = 0.9)”, “Lasso”, “Enet (alpha = 0.7)”, “StepCox (forward)”, “plsRcox”, “StepCox (both)”, “StepCox (backward)”, “RSF”, “GBM”. The TARGET dataset was severed as train sets, while the GEO was treated as the test set. The C-index was used to estimate the consistency of the model. The first was random forest with C-index up to 0.963, a mean of 0.79 (Figure 2).

Since the c-index value obtained by the random distribution forest method is the largest, we apply the var.select function meter to get the most important signaling pathways.

### Identification of key pathways by machine learning

Four pathways (PID IL2 STAT5 PATHWAY, REACTOME INTERLEUKIN 7 SIGNALING, WHITEHURST PACLITAXEL SENSITIVITY, YANG BREAST CANCER ESR1 UP) were identified through 20 machine learning. All the 4 pathways had been reported to be associated with osteosarcoma.

### Risk signature in TARGET and GEO datasets

To facilitate clinical application later, we applied a multifactorial Cox proportional risk regression model analysis to calculate the coefficients of the four signaling pathways.

**Figure 3:**
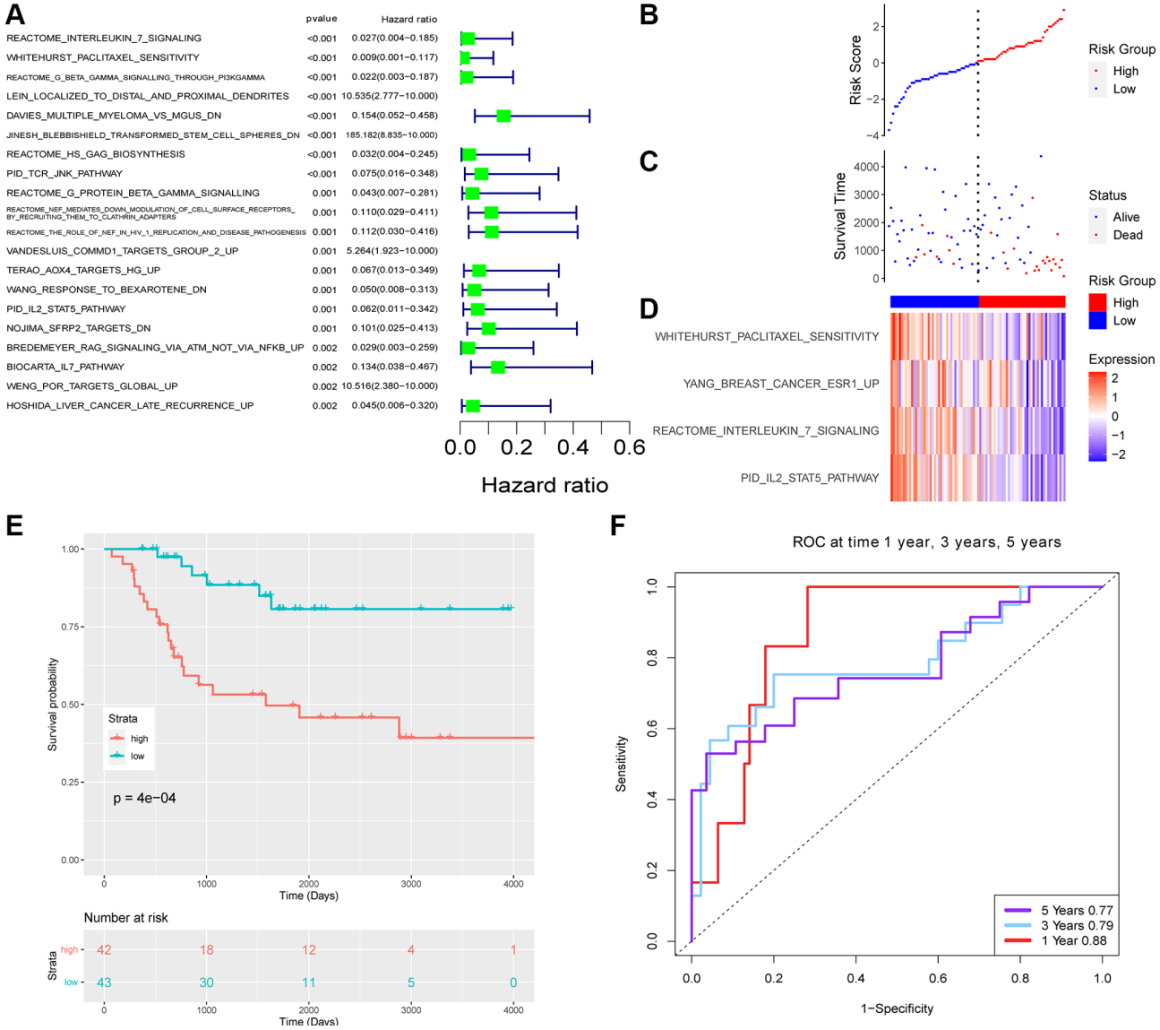
**The risk signature of TARGET datasets.** (**A**) Top 20 survival-related pathways with lowest *p*-value. Risk score (**B**) and survival time (**C**) distribution of TARGET. (**D**) The enrichment score of 4 pathways. (**E**) Survival analysis of two groups. (**F**) The ROC of the model.

**Figure 4:**
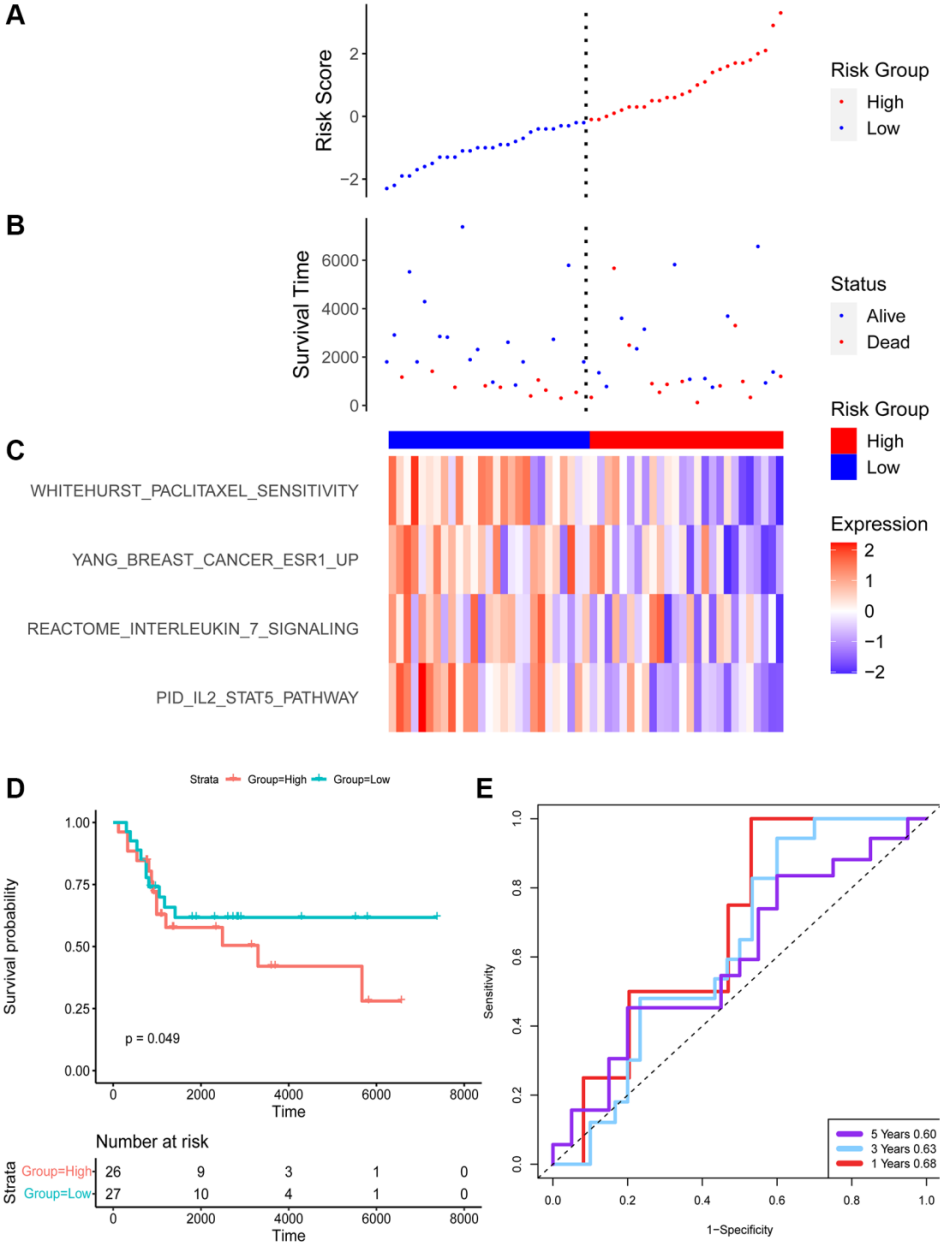
**The risk signature of GEO datasets.** Risk score (**A**) and survival time (**B**) distribution of TARGET. (**C**) The enrichment score of 4 pathways. (**D**) Survival analysis of two groups. (**E**) The ROC of the model.

The risk score was as following: Risk score = (−3.08) × (PID_IL2_STAT5_PATHWAY) + (−0.30) × (REACTOME_INTERLEUKIN_7_SIGNALING) + (−4.74) × (WHITEHURST_PACLITAXEL_SENSITIVITY) + (−2.14) × (YANG_BREAST_CANCER_ESR1_UP). According to the equate, the risk score distribution of the TARGET and GEO cohort was calculated (Figures 3 and 4).

Each patient with a risk score was divided into a low or high group. There was a significant difference in the survival of two groups. Patients in the low group had favored survival in both cohorts.

ROC of the two datasets was evaluated for 1, 3, and 5 years. The AUC of TARGET was 0.88, 0.79, and 0.77 for 1, 3, and 5 years respectively. The area under the AUC curve is slightly smaller for the GEO dataset with 0.68, 0.63, and 0.60 for 1, 3, and 5 years, respectively.

### Tumor microenvironment analysis

**Figure 5:**
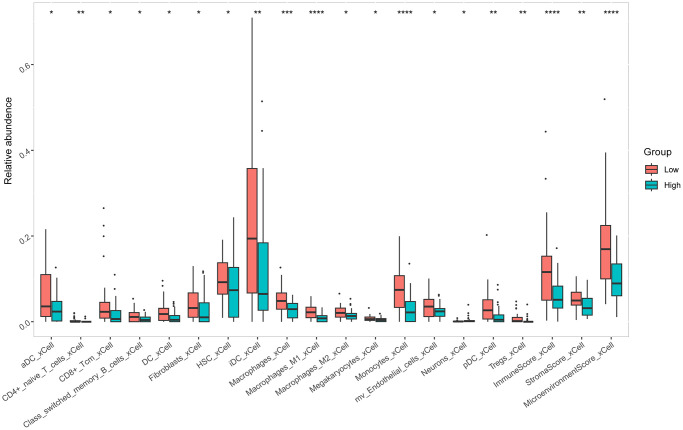
Differences of tumor immune infiltrating cells in osteosarcoma between two groups.

Tumor immune infiltration cells (aDC, CD4+ naive T cells, CD8+ Tcm, Class switched memory B cells, DC, Fibroblasts, HSC, iDC, Macrophages, Macrophages M1, Macrophages M2, Megakaryocytes, Monocytes, mv Endothelial cells, Neurons, pDC, Tregs, ImmuneScore, StromaScore, Microenvironment Score) were revealed for each patient. The Wilcox test was used to evaluate the difference of the two groups. To our surprise, statistical analysis showed differences between two groups of tumor immune infiltrating cells (Figure 5).

### Drug sensitivity analysis between high- and low-risk groups

**Figure 6:**
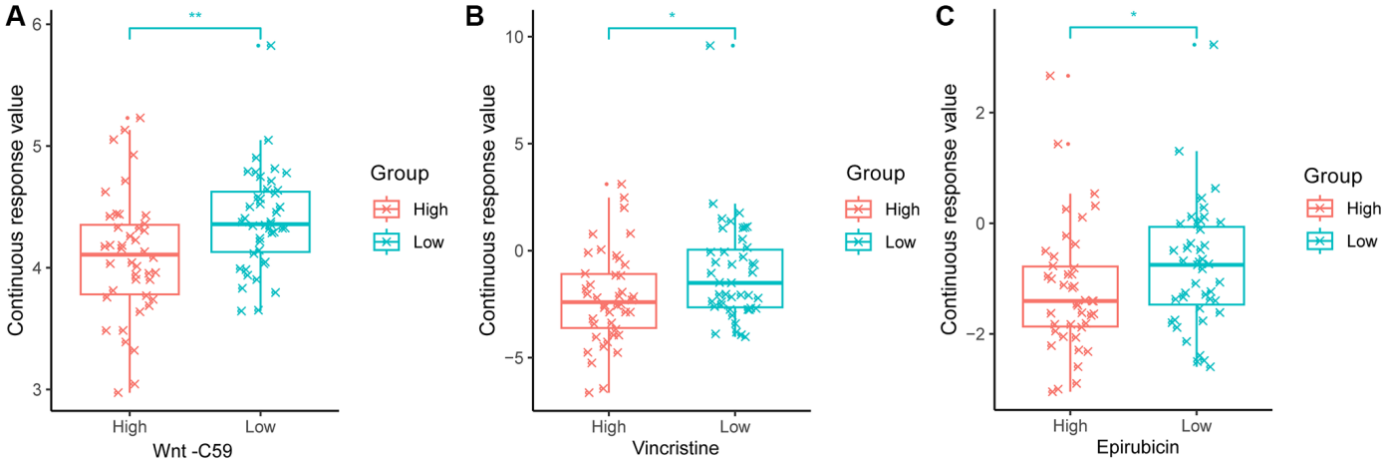
**Difference of three drugs' sensitivity of osteosarcoma.** (**A**) Wnt-C59. (**B**) Vincristine. (**C**) Epirubicin.

The relative drug sensitivity value was calculated for each patient based on Genomics of Drug Sensitivity in Cancer (GDSC) with 198 compounds or drugs. All the drug sensitivity valuse were shown in Supplementary Table 1. Figure 6 showed the presentive drugs for osteosarcoma patients. Patients in the high-risk group had more sensitivity in Wnt-C59, Vincristine, and Epirubicin (Figure 6).

## DISCUSSION

Osteosarcoma is a type of bone cancer originating in the bone formation cells. It is the most common primary bone cancer affecting children and young adults. Research has shown that genetic factors play a significant role in the development and progression of osteosarcoma. It is characterized by the rapid and aggressive growth of malignant cells that produce immature bone. Understanding the signaling pathways involved in osteosarcoma development and progression is crucial for identifying potential therapeutic targets and developing more effective treatments. Previous study had revealed some of the key osteosarcoma-related signaling pathways. For example, P53 and RB1 tumor suppressor pathways: Mutations in the TP53 and RB1 genes are frequently observed in osteosarcoma. These genes encode the p53 and retinoblastoma (Rb) proteins, respectively, which regulate cell cycle progression and apoptosis as tumor suppressors. Loss of function of these proteins due to genetic alterations leads to uncontrolled cell proliferation and contributes to osteosarcoma development. Wnt/β-catenin signaling pathway: The Wnt/β-catenin signaling pathway involves in various cellular processes, including cell proliferation, differentiation, and migration. Aberrant activation of this pathway has been implicated in osteosarcoma tumorigenesis. In osteosarcoma, increased expression of Wnt ligands and receptors, as well as genetic alterations in key pathway components, such as β-catenin and APC, can result in the stabilization and nuclear translocation of β-catenin, promoting the transcription of target genes associated with cell proliferation and survival [30, 31]. Notch signaling pathway: The Notch signaling pathway is another crucial regulator of cell fate determination and tissue homeostasis. Dysregulation of Notch signaling has been observed in various cancers, including osteosarcoma. In osteosarcoma, upregulation of Notch receptors and ligands, as well as mutations in pathway components, can lead to increased Notch signaling activity, promoting cell proliferation, survival, and metastasis. Hedgehog signaling pathway: The Hedgehog signaling pathway is involved in embryonic development and tissue patterning. Aberrant activation of this pathway has been implicated in several cancers, including osteosarcoma. In osteosarcoma, overexpression of Hedgehog ligands and receptors, as well as mutations in pathway components, such as SMO and PTCH1, can result in increased Hedgehog signaling activity, promoting tumor growth and metastasis.

Considering that tumorigenesis is a process in which multiple signaling pathways are involved, we calculated the enrichment scores of the signaling pathways by each patient. In this study, considering that tumorigenesis involves multiple signaling pathways, we calculated the enrichment scores of the signaling pathways by each patient. We identified 486 signal pathways that related to prognosis. The four most important signaling pathways (PID IL2 STAT5 PATHWAY, REACTOME INTERLEUKIN 7 SIGNALING, WHITEHURST PACLITAXEL SENSITIVITY, YANG BREAST CANCER ESR1 UP) were screened by 20 machine learning algorithms.

PID IL2 STAT5 PATHWAY, namely IL2 signaling events mediated by STAT5, was involved in multi-biological processes, including cell proliferation and stemness maintenance, etc., [32–34]. REACTOME INTERLEUKIN 7 SIGNALING mainly affects B cell, T cell, and NK cell growth, survival, and differentiation [35–38] and takes part in the drug sensitivity of osteosarcoma [39]. WHITEHURST PACLITAXEL SENSITIVITY affects drug sensitivity in osteosarcoma. Studies had shown paclitaxel inhibited the proliferation and promoted apoptosis [40].

These four signaling pathways are directly or indirectly associated with osteosarcoma biological behavior, suggesting that our approach is proper. To facilitate application in clinical practice, we constructed a multifactorial COX regression model using the four signaling pathways screened. The risk score of all the patients was calculated by the model. Each patient with a risk score was divided into low or high groups. There was a significant difference in the survival of two groups. Patients in the low group had favored survival in osteosarcoma.

The AUC of TARGET was 0.88, 0.79, and 0.77 for 1, 3, and 5 years, respectively, which suggested the model had good predictive value.

The tumor microenvironment can influence various tumor biological behaviors, especially immune cells within the tumor microenvironment. Therefore, we analyzed differences in tumor immune cells between the two groups according to the model. To our surprise, all tumor immune infiltration cells (aDC, CD4+ naive T cells, CD8+ Tcm, Class switched memory B cells, DC, Fibroblasts, HSC, iDC, Macrophages, Macrophages M1, Macrophages M2, Megakaryocytes, Monocytes, mv Endothelial cells, Neurons, pDC, Tregs, ImmuneScore, StromaScore, Microenvironment Score) were statistically different between the two groups. This result suggests a significant difference in the immune status of patients in the high- and low- risk groups, which means that our signature can not only predict the prognosis of patients but also the immune status of osteosarcoma patients, which has the potential value to be applied to predict the immune status of patients.

Furthermore, we analyzed drug sensitivity analysis between high and low risk groups. One hundred ninety-eight compounds or drug values were calculated based on the GDSC database. This result showed the model identified in the study can predict drug sensitivity.

Machine learning is revolutionizing the field of medical diagnosis and treatment, and it is also playing an increasingly important role in the diagnosis and treatment of bone cancer, specifically osteosarcoma. Osteosarcoma is a type of bone cancer that commonly affects children and adolescents, and early diagnosis is crucial for effective treatment and improved patient outcomes. In this context, machine learning algorithms have proved valuable tools in aiding the diagnosis and treatment of osteosarcoma.

One of the primary applications of machine learning in osteosarcoma is prognosis prediction in medical data analysis. Medical data, such as transcriptome, X-rays, magnetic resonance imaging (MRI), and computed tomography (CT) scans, play a crucial role in identifying tumors and evaluating their characteristics and prognosis. However, accurately interpreting these data can be a challenging task for clinicians. In this study, we used 20 machine learning algorithms to construct a prognostic prediction model with good prediction performance based on the enrichment score of pathways. Another area where machine learning is making a significant impact is in treatment planning. Osteosarcoma treatment decisions often involve a multidisciplinary team, including surgeons, oncologists, and radiation therapists. Machine learning algorithms can assist in analyzing patient data, such as medical history, genetic information, and treatment responses, to predict optimal treatment plans. By leveraging data from past cases and clinical trials, machine learning models can provide personalized treatment recommendations based on individual patient profiles. These models can consider various factors, including tumor characteristics, patient age, overall health, and treatment response rates, to guide the selection of appropriate therapies. This can increase the chances of successful treatment outcomes while minimizing potential side effects. Predictive treatment options were not addressed in this study, and we will explore this aspect of research in depth in future studies as well.

In summary, we identified a model consisting of four signaling pathways that not only predicts the prognosis of osteosarcoma patients but also the immune status of the tumor microenvironment as well as drug sensitivity. However, there are some limitations. For example, more datasets were needed to validate the signature and more experiments were needed to test those results.

## Supplementary Materials

Supplementary Table 1
